# Low mood, visual hallucinations, and falls – heralding the onset of rapidly progressive probable sporadic Creutzfeldt–Jakob disease in a 73-year old: a case report

**DOI:** 10.1186/s13256-018-1649-4

**Published:** 2018-05-08

**Authors:** Daniel Martin Klotz, Rose Sarah Penfold

**Affiliations:** 1 Department of Gynaecology and Obstetrics, University Hospital Dresden, Technische Universität Dresden, Fetscherstr. 74, 01307 Dresden, Germany; 2 Chelsea and Westminster Hospitals NHS Foundation Trust, West Middlesex University Hospital, Twickenham Rd, Isleworth, TW7 6AF UK; 3 Imperial College London, London, Kensington SW7 2AZ UK

**Keywords:** Creutzfeldt-Jakob disease, Geriatrics, Dementia, Falls, Neurological disorder

## Abstract

**Background:**

Creutzfeldt–Jakob disease is a rare and rapidly fatal neurodegenerative disease. Since clinicians may see only very few cases during their professional career, it is important to be familiar with the clinical presentation and progression, to perform appropriate investigations, and allow for quick diagnosis.

**Case presentation:**

A 73-year-old British Caucasian woman presented with acute confusion of 2 weeks’ duration on a background of low mood following a recent bereavement. Her symptoms included behavioral change, visual hallucinations, vertigo, and recent falls. She was mildly confused, with left-sided hyperreflexia, a wide-based gait, and intention tremor in her left upper limb. Initial blood tests, computed tomography, and magnetic resonance imaging of her brain showed no significant abnormality. Following admission, she had rapid cognitive decline and developed florid and progressive neurological signs; a diagnosis of prion disease was suspected. A lumbar puncture was performed; cerebrospinal fluid was positive for 14–3-3 protein, real-time quaking-induced conversion, and raised levels of s-100b proteins were detected. An electroencephalogram showed bilateral periodic triphasic waves on a slow background. The diagnosis of probable Creutzfeldt–Jakob disease was made.

**Conclusions:**

This case report highlights key features in the initial presentation and clinical development of a rare but invariably rapidly progressive and fatal disease. It emphasizes the importance of considering a unifying diagnosis for multifaceted clinical presentations. Although it is very rare, Creutzfeldt–Jakob disease should be considered a diagnosis for a mixed neuropsychiatric presentation, particularly with rapid progressive cognitive decline and development of neurological signs. However, to avoid overlooking early signal change on magnetic resonance imaging, it is important to take diffusion-weighted magnetic resonance imaging for all patients with neuropsychological symptoms. Importantly, early diagnosis also ensures the arrangement of suitable contamination control measures to minimize the risk of infection to health care professionals and other patients.

## Background

Creutzfeldt–Jakob disease (CJD) is a rare neurodegenerative disease caused by a misfolding of a host protein called a prion protein (PrP). When a pathologically misfolded PrP (PrP^Sc^) is formed, it acts as a seed for the misfolding of normal cellular PrP (PrP^C^). This causes a chain reaction which leads to cell death and the manifestations of the disease [1]. Most commonly it occurs sporadically (sCJD), but may also be genetic or variant (acquired) in etiology. All forms are rapidly progressive and invariably fatal, with sCJD affecting 1 to 2 per million people per year worldwide [2]. As PrP is most abundant in neurons, the early manifestations are neurological [1].

Neither a cure nor disease-modifying treatments exist for suspected cases of CJD. However, early suspicion facilitates relevant diagnostic investigations, allowing for discussion with patients, family, and a specialist clinical unit in a timely manner and for supportive management. Specific infection control procedures should be put in place for all invasive procedures when CJD is suspected as a potential diagnosis [3]. Case detection is vital in disease surveillance and monitoring; case detection is also important for research into improving diagnosis and toward future treatment. Reviewing this case, the presentation and clinical progression corroborated with many other cases documented in the literature. However, the diagnosis was not initially suspected, perhaps because most physicians do not encounter a case in the entirety of their career and many of the symptoms are nonspecific: a depressive prodrome, memory loss, and acute confusion are common in the elderly on the acute medical take, and the differential for cerebellar signs is broad. The rapid progression of neurological signs may increase suspicion of a potential diagnosis of CJD.

This could be considered a good example of the Law of Diagnostic Parsimony (Occam’s razor): although multifactorial causes of presenting complaints are common in the elderly, sometimes there is just one underlying diagnosis to account for all symptoms.

## Case presentation

Our patient, a previously well and independent 73-year-old Caucasian woman, presented to our Emergency department (ED) with subacute onset confusion. Her family reported worsening confusion in the preceding 2 weeks, which was associated with behavioral changes that were mainly related to visual hallucinations, waking up at night and talking to herself, looking for her late sister, and thinking that there were men around her bed. She had started experiencing vertigo and had fallen at home 2 weeks prior to admission, although she denied associated head injury. The onset of symptoms reportedly coincided with a family bereavement and our patient had recently been restarted on antidepressants by her general practitioner (GP) due to pervasive low mood. She had no infective symptoms. Her past medical history included hypertension, depression, and anxiety. Prior to admission, she lived alone and independently, with no concerns regarding cognition. She was taking prochlorperazine (vertigo), doxazocin, and paroxetine (depression) confirmed by her GP and Pharmacy medicines reconciliation; she denied any other previous or current drug or alcohol use and this was corroborated by her family. She did not smoke tobacco. She had no significant familial history and no children.

On admission she appeared alert, Glasgow Coma Scale (GCS) 15/15. Her Abbreviated Mental Test score (AMTS) on admission was 9/10. She was moderately hypertensive (160/63), with all other observations within normal range. Cardiovascular, respiratory, and abdominal examinations were unremarkable. However, abnormalities were seen on neurological examination. She appeared unsteady on standing with a wide-based gait, falling to the left side on attempted mobilization. Reflexes in her lower limbs were noted to be brisk (left>right) and on examination of her upper limbs there was left-sided intention tremor, dysdiadochokinesis, and past-pointing. Overall it was a predominantly cerebellar picture, with some upper motor neuron features.

Initial investigations were grossly normal (see below); she was admitted to our medical ward for further observation and investigations. During initial admission, a broad range of concurrent differential diagnoses were suggested to account for her recent behavioral changes and ataxia.

**Fig. 1:**
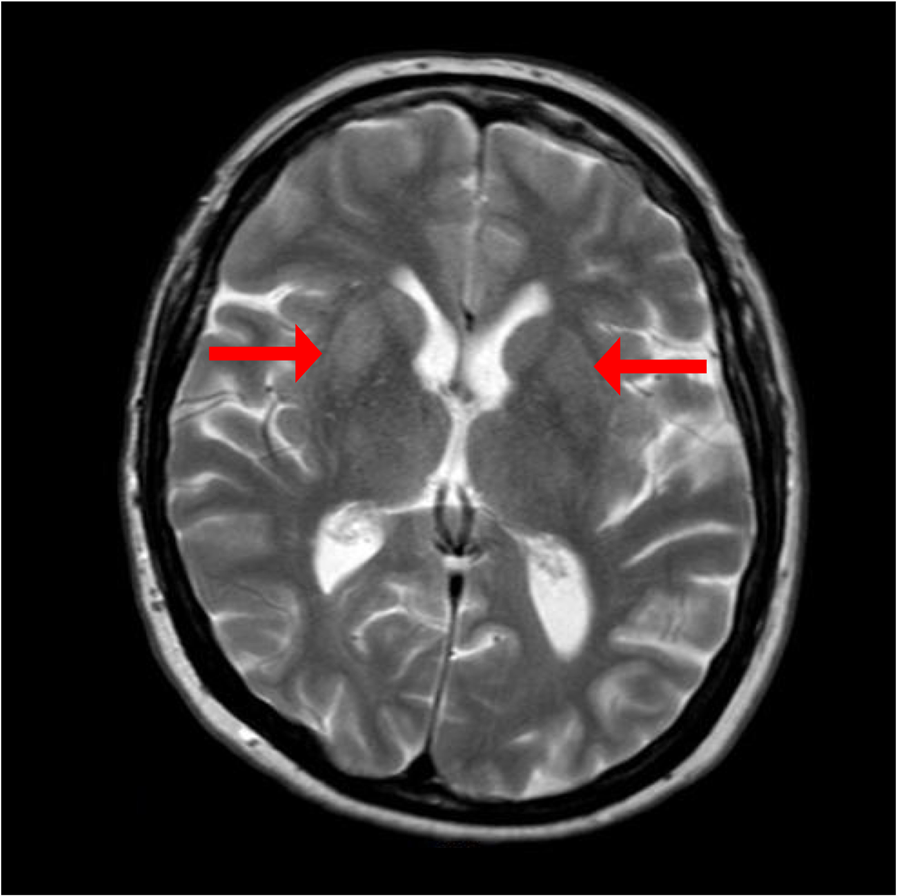
Magnetic resonance image of the patient during admission. T2-weighted magnetic resonance image showing bilateral symmetrically increased signal intensity in the basal ganglia (*red arrows*)

On admission, blood tests showed a mildly raised C-reactive protein (CRP) of 9.8 mg/L and mildly raised urea; otherwise a full blood count (FBC), urea and electrolytes (U&Es), bone profile, liver function tests (LFTs), and thyroid function tests (TFTs) were all within normal range. Specifically: white cell count (WCC) 6.0 × 10^9^/L (reference range 3.8–10.8), hemoglobin (Hb) 126 g/L (117–155), platelets 220 × 10^9^/L (140–400), sodium (Na) 135 mmol/L (136–146), potassium (K) 4.1 (3.5–5.1), creatinine 73 umol/L (45–84), urea 9.0 mmol/L (2.8–7.2), calcium (Ca) 2.42 mmol/L (2.2–2.65), inorganic phosphate 1.14 mmol/L (0.81–1.45), and alkaline phosphatase (ALP) 98 IU/L (30–120); albumin 40 g/L (35–52) and CRP 9.8 mg/L (0–7.9). A urine dipstick was negative and there was no growth from a midstream urine (MSU) test. A chest X-ray (CXR) revealed no abnormality. A computed tomography (CT) scan of her head showed no acute intracranial abnormality. On the initial magnetic resonance imaging (MRI) report, a few small nonspecific white matter lesions were noted, no intracranial abnormality correlating with our patient’s symptoms was identified. No comment was made on the thalamus or basal ganglia. Following later specialist review, this MRI scan was thought to be consistent with prion disease, with bilateral symmetrically increased signal intensity in the basal ganglia on T2-weighted imaging (Fig. 1).

Electroencephalography (EEG) showed a background of intermixed delta activity at rate 2.5–3.9 Hz and theta activity at rate 4–5.5 Hz bilaterally through all channels, interrupted by prolonged runs of anteriorly dominant widespread triphasic waves – bilateral independent periodic lateralized epileptiform discharges (BiPLEDs) – occurring at 1–2 Hz and lasting from several seconds to a few minutes. These findings were reported as suggestive of bilateral cerebral/cortical dysfunction, with possible causes including prion disease, viral encephalitis, metabolic, and vascular.

A lumbar puncture was performed with the following results: cytology – no cells identified; viral polymerase chain reaction (PCR) – herpes simplex virus (HSV) deoxyribonucleic acid (DNA), varicella zoster virus (VZV) DNA, enterovirus RNA not detected; oligoclonal bands – cerebrospinal immunoglobulin G (IgG) 32 mg/L (10–40); cerebrospinal fluid (CSF) protein 0.5 g/L (reference range 0.15–0.8); and CSF glucose 3.8 mmol/L (2.2–3.9). A CSF sample was sent to the National CJD Research & Surveillance Unit (NCJDRSU) in Edinburgh, which was found to be positive for 14–3-3 protein and real-time quaking-induced conversion (RT-QuIC) was positive. Raised levels of s-100b protein > 2.5 ng/mL (reference range < 0.41 ng/mL) were detected.

A definitive diagnosis of CJD is neuropathological, requiring brain biopsy or post-mortem analysis. Our patient’s family did not consent to post-mortem analysis and declined further genetic testing for familial CJD.

Within weeks following her admission to the ward, she underwent a rapid clinical deterioration. After 2 weeks she was unable to follow simple verbal commands, with worsening hypertonia bilaterally (although always more marked on the left than the right). There was difficulty following simple instructions and speech became slower and more fragmented. She developed abnormal eye movements, with hypometric saccades and impersistence of gaze. Her left arm was noted to be akinetic with abnormal posturing and spontaneous myoclonus, as well as hyperreflexia.

A month following admission, she was akinetic, mute, and unresponsive to external stimuli. She had extensive secretions requiring suctioning and was nasogastric tube fed with high risk of aspiration. Following discussion with her family and the palliative care team, a decision was made to start a compassionate care agreement and she died the following week.

## Discussion and conclusions

Our patient’s initial presentation was a complex and mixed neuropsychiatric picture. During her clerking, common causes were considered to account for each symptom individually (an application of Hickam’s dictum: it is often more likely that a patient has several common diseases rather than having a single rare disease to explain a myriad of symptoms). Frequent causes of confusion in the elderly – infection, drug/alcohol intoxication or withdrawal, metabolic or vascular – were all investigated with blood tests and scans and a psychiatry opinion sought relating to her known diagnosis of depression. Common causes of cerebellar symptoms – alcohol use, vascular – were appropriately investigated. However, our patient’s family did not report an alcohol history, and many of the differentials explored would not have accounted for all of her psychiatric, cognitive, and neurological symptoms.

Following admission, she was started empirically on acyclovir for herpes simplex encephalitis. Following EEG, this was still considered a differential but the neurological signs seen here would have been an unusual presentation. In addition, the CSF did not support this diagnosis.

With EEG and specialist interpretation of the MRI, CJD was the most likely diagnosis to account for all of her symptoms. Probable diagnosis of sCJD was further confirmed with supporting CSF findings: positive for 14–3-3 protein, raised protein levels of s-100b > 2.5 ng/mL (reference range < 0.41 ng/mL), and a positive RT-QuIC. The clinical review by the NCJDRSU further supported the diagnosis. It should be noted that in practice, the most commonly used clinical criteria for probable sCJD do not help with early diagnosis of CJD as akinetic mutism and the characteristic EEG changes often do not occur until late stages of the illness [4]. Furthermore, the evidence to support use of ancillary tests (such as EEG and CSF 14–3-3 protein) is mixed. Such tests have been demonstrated to have poor sensitivity and/or specificity and are often not useful in clinical practice [5].

CJD has attracted extensive scientific and public interest due to its rare pathogenicity and notorious media coverage of the variant form (vCJD); however, the incidence is very low and early symptoms are often nonspecific, leading to frequent delay in appropriate investigations and diagnosis. Initial presentation may be psychiatric, neurological, or mixed; this patient’s psychiatric prodrome followed by rapid development of neurological symptoms is corroborated by other case reports [6, 7]. The first symptom of CJD is usually rapidly progressive dementia, leading to memory loss, personality changes, and hallucinations. When considering a rapidly progressive dementia, particularly a patient with prominent motor and/or cerebellar dysfunction, CJD should be high on the list of differential diagnoses [8].

Previously, sCJD has been characterized by prominent neurological symptoms, with vCJD more psychiatric in presentation and course. However, a retrospective review of 126 cases of sCJD found that 80% of cases demonstrated psychiatric symptoms (depression, anxiety, psychosis, behavior change, sleep disturbance) within the first 100 days of illness, with 26% occurring at presentation [9]. Psychiatric manifestations may be an early and prominent feature of sCJD, often occurring prior to formal diagnosis; however, as in this case, psychiatric manifestations may be misattributed to more common mood disorders or other causes of cognitive impairment in the elderly. Other less common initial presentations such as the Heidenhain visual variant have also been reported [10].

sCJD is classified according to the following internationally recognized criteria updated in 2010, although further modifications have been proposed [11]:

- Possible – rapidly progressive dementia plus two of: myoclonus, visual or cerebellar problems, pyramidal/extrapyramidal features, akinetic mutism.
- Probable – meets possible criteria plus one or more of: typical EEG, high signal in caudate/putamen on MRI or positive 14–3-3 protein in CSF.
- Definite – neuropathological or immunohistochemical confirmation.

This case was probable, with a rapidly progressive dementia and all other associated symptoms as well as MRI, EEG, and CSF abnormalities (no neuropathology specimen). Characteristic brain MRI lesion patterns are the most recent addition to these criteria and are helpful in establishing a diagnosis of sCJD, with high sensitivity and specificity for the disease [12]. However, a major limitation is that CJD-associated MRI changes are often not documented on the formal investigation report from the referring center. A recent study by the National Prion Centre found that specialists were able to identify sCJD-associated MRI changes in 83/91 cases (91% sensitivity), whereas referring centers documented CJD-associated MRI changes in 43 of the cases (47% sensitivity) [13]. In this case, the possibility of prion disease was not raised on the initial MRI request and not reported by a non-specialist. Specialist review by an experienced neuroradiologist or prion disease specialist unit could facilitate more rapid diagnosis. CT may reveal evidence of mild brain atrophy in some cases but this remains uncommon due to the speed of onset of CJD.

Almost all cases of CJD show altered EEG signals. Slow wave activity remains constant and deteriorates with the progression of the disease. Pseudo-periodic sharp wave complexes of 1 Hz are characteristic of EEG in cases of sCJD [14]. The presence of protein 14–3-3 is also a specific finding, particularly concomitant with EEG changes. A positive RT-QuIC and raised s-100b protein levels further support the diagnosis of probable CJD [15]. Although new tests have greatly enhanced the sensitivity and specificity of diagnosing CJD, other CSF biomarkers are currently being investigated [1, 16].

Case reports such as this are vital in raising awareness of the clinical presentation and course of this rare neurodegenerative disorder. Clinical suspicion facilitates appropriate investigations and referral to the NCJDRSU, guiding further management and research. In particular, scientific and clinical advances in disease-modifying treatment requires cases to be detected before they become clinically advanced. Although early diagnosis does not alter the outcome of the disease, it allows clinicians to initiate early advance care planning (ACP) discussions with patients and their families. There is generally a low public awareness but a high expressed willingness to engage in an ACP discussion [17]. ACP can facilitate the delivery of care more in keeping with a patient’s wishes and increase patient and family satisfaction with care [18].

In a previously well patient presenting with a myriad of new-onset signs and symptoms, it is vital to remember Occam’s razor and consider a unifying diagnosis.

## Abbreviations

ACP: Advance care planning
ALP: Alkaline phosphatase
AMTS: Abbreviated Mental Test score
BiPLEDs: Bilateral independent periodic lateralized epileptiform discharges
Ca: Calcium
CJD: Creutzfeldt–Jakob disease
CRP: C-reactive protein
CSF: Cerebrospinal fluid
CT: Computed tomography
CXR: Chest X-ray
DNA: Deoxyribonucleic acid
ED: Emergency department
EEG: Electroencephalography
FBC: Full blood count
GCS: Glasgow Coma Scale
GP: General practitioner
Hb: Hemoglobin
HSV: Herpes simplex virus
IgG: Immunoglobulin G
K: Potassium
LFTs: Liver function tests
MRI: Magnetic resonance imaging
MSU: Midstream urine
Na: Sodium
NCJDRSU: National CJD Research & Surveillance Unit
PCR: Polymerase chain reaction
PrP: Prion protein
PrP^C^: Normal cellular prion protein
PrP^Sc^: Pathologically misfolded, prion protein scrapie
RT-QuIC: Real-time quaking-induced conversion
sCJD: Sporadic Creutzfeldt–Jakob disease
TFTs: Thyroid function tests
U&Es: Urea and electrolytes
vCJD: Variant Creutzfeldt–Jakob disease
VZV: Varicella zoster virus; WCC, White cell count
